# Flavonoids contribute most to discriminating aged Guang Chenpi (*Citrus reticulata* ‘Chachi’) by spectrum‐effect relationship analysis between LC‐Q‐Orbitrap/MS fingerprint and ameliorating spleen deficiency activity

**DOI:** 10.1002/fsn3.3629

**Published:** 2023-09-21

**Authors:** Xiaoming Sun, Haidan Deng, Baojun Shan, Yunqi Shan, Jiaying Huang, Xinshu Feng, Xiaomin Tang, Yuewei Ge, Peiran Liao, Quan Yang

**Affiliations:** ^1^ School of Pharmacy Guangdong Pharmaceutical University Guangzhou China; ^2^ Key Laboratory of Production & Development of Cantonese Medicinal Materials State Administration of Traditional Chinese Medicine Guangzhou China; ^3^ Guangdong Provincial Research Center on Good Agricultural Practice & Comprehensive Agricultural Development Engineering Technology of Cantonese Medicinal Materials Guangzhou China; ^4^ Comprehensive Experimental Station of Guangzhou Chinese Material Medica, China Agriculture Research System (CARS‐21‐16) Guangzhou China; ^5^ School of Traditional Chinese Medicine Guangdong Pharmaceutical University Guangzhou China

**Keywords:** *Citrus reticulata* ‘Chachi’, flavonoids, gut microbiota, spleen deficiency, traditional Chinese medicine

## Abstract

To further explore the mechanism of “the longer storage time, the better bioactivity” of aged Guang Chenpi, the dry pericarp of *Citrus reticulata* ‘Chachi’ (CRC), a series of activity assessments were performed on spleen deficiency mice. The constituents in CRC with different storage years were analyzed by LC‐Q‐Orbitrap/MS. A total of 53 compounds were identified, and CRC stored for more than 5 years showed higher flavonoid content, especially that of polymethoxyflavones. Anti‐spleen deficiency bioactivity analysis among various CRC with different storage years showed aged CRC (stored for more than 3 years) could significantly alleviate fatigue and depression behaviors much better, increase D‐xylose and gastrin secretion, and upregulate the expression of the linking protein occludin in the colon walls. Results from 16S rDNA sequencing showed that aged CRC could downregulate the abundance of *Enterococcus*, *Gemmata*, *Citrobacter*, *Escherichia_Shigella*, and *Klebsiella*, which were significantly overrepresented in the model group. *Bacteroides*, *Muribaculum*, *Alloprevotella*, *Paraprevotella*, *Alistipes*, *Eisenbergiella*, and *Colidextribacter* were downregulated in the model group but enriched in the CRC groups. At last, the spectrum‐effect relationship analysis indicated that flavonoids such as citrusin III, homoeriodictyol, hesperidin, nobiletin, and isosinensetin in aged CRC showed the highest correlation with better activity in ameliorating spleen deficiency by regulating gut microbiota. Flavonoids contribute most to discriminating aged CRC and could disclose the basis of “the longer storage time, the better bioactivity” of aged Guang Chenpi.

## Graphical abstract

We found that aged Guang Chenpi could rescue spleen deficiency by ameliorating gut microbiota dysfunction and fatigue and depression behaviors, upregulating D‐xylose and gastrin secretion, and increasing the integrity of the colon tissue better than a year‐old sample. And these rescuing effects were associated with citrusin III, homoeriodictyol, hesperidin, nobiletin, and isosinensetin in samples with different storage years. Our findings will serve as a reference for the comprehensive evaluation of the mechanism of “the longer storage time, the better bioactivity” in the processing of Guang Chenpi.

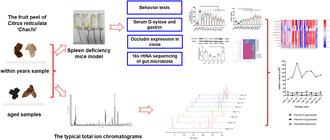

## 1 INTRODUCTION

“The longer storage time, the better bioactivity” is known as “Chen Jiu Zhe Liang” in Chinese, which means that herbal medicine should be stored for many years before being used in the clinic (Nijat et al., 2022). This is one of the important methods of preprocessing traditional Chinese medicine that has been recorded for many Chinese medicines (Luo et al., 2019). Guang Chenpi is the dry pericarp of the fruits of *Citrus reticulata* “Chachi” (CRC) that is mainly planted in Guangdong province. It is recorded in the Chinese Pharmacopeia (2020 edition) to exert bioactivities such as regulating qi and strengthening the spleen, drying dampness, and eliminating phlegm (Yu et al., 2018). Guang Chenpi is also widely used as a medicinal food in South China and other Asian countries (Xu et al., 2014). Currently, the market price of Guang Chenpi is related to its storage time. However, there is not enough evidence to confirm the relationship between its bioactivity and extended storage time. Therefore, studying the correlation between bioactivity and extended storage time may explain the mechanism of the traditional uses of “Chen Jiu Zhe Liang.”

The chemical constituents of Guang Chenpi mainly include flavonoids, volatile oils, alkaloids, and polysaccharides (Yu et al., 2018). Evidence indicates that flavonoids and volatile oils can exert liver protection, hyperglycemia, pancreatic protective, and anticancer effects owing to their anti‐inflammatory, antioxidant, antibacterial, and glycosidase‐inhibiting effects (Bhandari et al., 2021; Elhelaly et al., 2019; Manthey et al., 2001; Zeng et al., 2018). The flavonoid glycoside and polymethoxyflavonoid content of Guang Chenpi obtained from different areas of cultivation and after varying harvest times differ considerably (Zheng et al., 2021). Furthermore, some studies show that its polymethoxylated flavone and antioxidant content increase with an increase in storage years (Fu et al., 2017; Zeng et al., 2017). Headspace‐solid phase microextraction combined with GC–MS has been used not only to analyze flavonoids but also the volatile oil components in Citri Reticulatae Pericarpium (CRP) after different harvest times (Zheng et al., 2018). Furthermore, “activity fingerprints” have been established for Chenpi samples from different cultivars (Zheng, Chao, et al., 2020), and near‐infrared spectroscopy has been used to establish a qualitative analysis model of CRP to distinguish between samples from different origins (Li et al., 2020). The DNA barcoding method has also been used to effectively distinguish Guang Chenpi and CRP from other related species. It provides a molecular basis for the identification of medicinal materials (Liu et al., 2021). The above evidence shows that chemical analysis can reveal the components that are present; however, studies on their correlation with the bioactivity of Guang Chenpi are lacking. Therefore, it is meaningful to develop an accurate method to explore the changes in bioactivity resulting from different years of storage.

Long‐term and extensive clinical applications of Guang Chenpi reveal that it ameliorates spleen deficiency‐related diseases, including indigestion, lack of appetite, abdominal fullness, and distention (Yu et al., 2018). Its mechanism of alleviating spleen deficiency has been reported to be related to the modulation of the gut microbiota. 16S rDNA gene sequencing shows that CRP can restore the abundance of gut microbiota in rats with spleen deficiency not only by upregulating some of the short‐chain fatty acid‐producing and anti‐inflammatory bacteria but also by downregulating certain spleen deficiency‐aggravating bacteria (Zheng, Zeng, et al., 2020). CRP also modulates the immune system (Zhou et al., 2021). CRP flavonoids can modulate the mRNA expression of gastric hormones CD3~+ and TFF3 in rats with spleen deficiency.

Although a few studies on active differences between aged and within‐year Guang Chenpi have been reported, no data are available on whether the aged samples have better activities. Hene, the gut microbiota composition, gastric hormone levels, and normal behaviors of rats were determined in this study. The chemical spectra and component differentiations of Guang Chenpi samples stored for 1–36 years were analyzed. The model of spectrum‐effect relationship established in this study will pave the way to exploring the theoretical mechanism of “Chen Jiu Zhe Liang” in the preprocessing of Guang Chenpi.

## 2 MATERIALS AND METHODS

### 2.1 Guang Chenpi sample collection and preparation

Guang Chenpi (*Citrus reticulata* ‘Chachi’) was collected from the Xinhui area in the Guangdong Province and identified by Professor Yang Quan at Guangdong Pharmaceutical University, Guangzhou, China. The storage period ranged from 1 to 36 years.

Smashed CRC samples (passed through a 40‐mesh sieve) were boiled with water and filtered twice. The filtrates were combined and concentrated to 1 g/mL. The positive control drug, Shen Ling Bai Zhu powder, was dissolved in water to yield a concentration of 1.5 g/mL (Luo et al., 2022). The aqueous extract of senna leaf was boiled in water for 5 min in a 1:5 ratio of material: liquid, filtered, and concentrated to 1.5 g/mL (Ma et al., 2019).

### 2.2 LC–MS analysis

The chemical constituents of Guang Chenpi water extract were analyzed using high‐performance liquid chromatography (HPLC) and a Waters Symmetry C18 column (4.6 mm × 250 mm, 5 μm). The mobile phases were aqueous 0.1% formic acid (A) and acetonitrile (B). The gradient elution was as follows: 0–10 min, 10%–25% B; 10–20 min, 25%–60% B; 20–30 min, 60%–95% B. The flow rate was 1 mL/min, the column temperature was 30°C, and the injection volume was 3 μL. Mass spectrometry (MS) was performed using a Q Exactive Focus mass spectrometer (Thermo Fisher Scientific Co., Waltham, USA) combined with an electrospray ion source and positive and negative ion switching scanning modes. The scanning range was 150–2000 m/z, the full MS primary resolution was 35,000, and the secondary resolution was 17,500. The ion‐spray voltage was 3.5 kV, the ion‐source temperature was 320°C, the sheath gas flow rate was 35 arb, the auxiliary gas flow rate was 15 arb, and the ion‐transport tube temperature was 320°C. The collision energy gradient was 20, 40, and 60 eV. Identification of compounds was done using Compound Discoverer software (version 3.0), and the original data were matched with mzCloud and the Chinese Medicine Compounds (OTCML) database.

### 2.3 Establishment of the mouse model of spleen deficiency

An equal number of 6–7‐week‐old male and female SPF‐grade Swiss mice weighting 20–25 g were purchased from Guangdong Medical Laboratory Animal Center (SCXK2018‐0002). All mice were housed at a constant temperature (26 ± 2°C) and standard humidity and subjected to appropriate light/dark conditions. Mice were allowed free access to water. All procedures were approved by the Institutional Animal Use Committee (Ethics Committee of GDPU on Laboratory Animal Care, No. gdpulacspf2017448).

All mice were randomly divided into either a control group (Control), a spleen deficiency model group (Model), a CRC group with different storage years (CRC‐1, CRC‐3, CRC‐5, CRC‐7, CRC‐9, CRCY‐15, CRC‐23, CRC‐36), or a positive control group (SLBZ) (*n* = 8 per group). Except for those in the control group, all other mice were administered aqueous senna extract intragastrically (*i.g*. 0.2 mL/20 g). Mice in the control group received a similar volume of normal saline by gavage for 14 days. Mice in each group were forced to swim once daily in a water tank for 14 consecutive days. The water temperature was 25°C, and the tank depth was 30 cm. Moreover, mice in each group were fasted every other day for 14 days (Zhang et al., 2021). Mice in the control and model groups were administered normal saline (*i.g*. 0.2 mL/20 g), whereas those in the CRC groups received a corresponding dose of CRC extract (*i.g*. 2 g/kg) and Shen Ling Bai Zhu powder for 14 days. The administered dose is calculated as 6–9 g for one person per day, according to the Chinese Pharmacopeia (2020 edition).

### 2.4 Behavior test

#### 2.4.1 Sugar preference test

After adaptive sugar‐water training, two identical water bottles of equal volumes were placed in each cage. For the first 24 h, 1% sucrose water was filled into both water bottles. Then, the solution in one of the bottles was replaced with pure water, whereas the contents of the other bottle were not changed. The sugar–water preference test was conducted on mice in each group on the 7th day after administration. Mice in each group were fasted for 24 h before the formal experiment. The consumption volume of sugar and pure water for 1 h was recorded (Zhang, Niu, Zhang, et al., 2022). The sugar water preference rate (W%) = sugar water intake/(sugar water intake + pure water intake) × 100%.

#### 2.4.2 Tail‐suspension test

The tail‐suspension test was performed after 8 days of drug administration. Briefly, the tail of each mouse was fixed on top of an iron platform such that the mouse was inverted and suspended with its head approximately 15 cm from the ground. Mice were observed for 6 min, and the activity in the last 4 min was recorded. The immobility time of each mouse was calculated (Yang et al., 2022).

#### 2.4.3 Forced‐swimming test

After drug administration for 9 days, mice were forced to swim in a 15‐cm‐deep glass beaker for 6 min. The 6 min for which they floated were recorded, and the duration of immobility for the last 4 min was calculated (Zhu et al., 2022).

#### 2.4.4 Open‐field test

The open‐field test (OFT) was performed using a KW‐OPF open‐field activity box (Guangzhou Bite Biotechnology Co., Ltd., Guangzhou, China) after 10 days of drug administration. Mice were gently placed in an open box with black walls and a bottom. The test was conducted in absolute silence and without any direct light source. Behavioral changes within 5 min, including the total distance and the time spent in the central area (25 × 25 cm), were recorded using a synchronous camera system (Hu et al., 2022). After each experiment, 75% ethanol was sprayed on the bottom and surrounding surfaces of the open‐field activity box and dried with a paper towel to avoid the influence of the residual odor of rats on subsequent tests involving a different batch of rats.

### 2.5 Determination of D‐xylose and gastrin levels

For the determination of serum D‐xylose levels, mice were fasted for 12 h before the *i.g*. administration (5%, 0.5 mL) of D‐xylose solution. Serum from mice was collected after 30 min. Mice were anesthetized using 1.25% avertin, and their whole blood was collected after removing their eyeballs. The blood was allowed to stand for 4 h at 4°C. The upper layer of serum was collected for analysis. Serum D‐xylose (20210823, Nanjing Jiancheng Bioengineering Institute, Nanjing, China) and gastrin (202104, Wuhan Aidi Biotechnology Co., Ltd., Wuhan, China) levels were determined using the phloroglucinol method according to the manufacturer's instructions.

### 2.6 Determination of intestinal propulsive ratio and gastric residual rate

Mice were administered a mixture of activated carbon and 10% sodium carboxymethyl cellulose. After 1 h, they were anesthetized using 1.25% avertin. Blood was collected, the pylorus was ligated, and the mesentery was stripped. The intestine from the pylorus to the ileocecal junction was cut and gently placed on the tray in a straight line. The total length of the small intestine was measured, and the distance from the pylorus to the furthest point to which the carbon had advanced in the intestine was recorded (Deng et al., 2018). The intestinal propulsive ratio was calculated as the distance traveled by carbon in the intestine/the total length of the small intestine × 100%. The gastric residual rate was calculated as follows: (total weight of the stomach−net weight of the stomach)/weight of the carbon paste × 100%.

### 2.7 16S rDNA gene sequencing of fecal samples

The DNA from the samples was extracted using an E.Z.N.A.® Stool DNA kit according to the manufacturer's instructions. Total DNA samples were diluted with 50 μL elution buffer and stored at −80°C until a polymerase chain reaction was performed (LC‐BioTechnology Co., Ltd.). The V4 regions of 16S rDNA were amplified using 341F (5′‐CCTACGGGNGGCWGCAG‐3′), 805R (5′‐GACTACHVGGGTATCTAATCC‐3′). Samples were sequenced on the NovaSeq PE250 platform according to the standard protocols. Sequencing data were processed using the Quantitative Insights Into Microbial Ecology (QIIME, v1.8.0) software.

### 2.8 Histopathology analysis

Colon tissues were immersed in 4% paraformaldehyde to fix for dehydration and then paraffin‐embedded and sectioned for hematoxylin and eosin (H&E) staining. Pathological changes were observed using the OLYMPUS/BX fluorescence microscope (Guangzhou Zhongchuang Biotechnology Co., Ltd., Guangzhou, China).

### 2.9 Western blotting

The total protein of colon tissue was extracted by adding a RIPA lysis solution containing a phosphatase inhibitor and protease inhibitor (1:100). The total protein concentration was determined using a bicinchoninic acid assay kit (BCA) (P0012S, Beyotime Biotechnology, Shanghai, China). After quantification, 40 μg protein was used for the electrophoretic separation on a 10% separation gel. Next, the protein was transferred to a polyvinylidene fluoride membrane. After blocking, they were incubated at 4°C with the primary antibody occludin (1:10,000, ab167161, Abcam, English) for 12 h. After washing three times with TBST solution (10 min/wash), the secondary antibody (1:10,000, NO. 7074S, Cell Signaling Technology, USA) was added and further incubated at 26°C for 1 h. An enhanced chemiluminescence chromogenic solution was added for imaging, and the gray value of the bands was measured using ImageJ software.

### 2.10 Gray relational analysis (GRA)

GRA is a method to study the degree of similarity or dissimilarity of development trends among factors. It is used to measure the degree of association between different factors and to evaluate the degree of correlation between the components and pharmacological effects of traditional Chinese medicine (Xiao et al., 2022; Yang et al., 2022; Zhang, Yu, Yang, et al., 2022). In this study, pharmaceutical factors correlating with spleen deficiency from CRC samples with different storage years were used as the mother sequence, and each characteristic peak quantification index was used as the sub‐sequence. The original data were dimensionless and processed using the mean value method. The resolution coefficient (ξ) was 0.5, and the gray correlation coefficients and correlation magnitudes were calculated (Qi et al., 2022). At the same time, the mean value was also used to calculate a composite score for spectral efficiency correlation, and the scores were ranked. By analyzing the magnitude of correlation between compounds and pharmacological effects, the contribution of each characteristic peak of CRC to ameliorating spleen deficiency symptoms was determined.

### 2.11 Statistical analysis

Data were analyzed using GraphPad Prism, and the results are expressed as the mean ± standard error of the mean. Statistically significant differences were analyzed using one‐way analysis of variance (ANOVA) with Tukey's post hoc test. *p* < .05 was considered significant. Compound Discoverer 3.0 software was used to accurately fit the molecular formula and primary mass spectrum of compounds. The results were matched with those in the mzCloud network database and the OTCML database, and the chromatographic peaks were determined. To further speculate on and identify the structure of compounds, the ion information of the primary and secondary segments of the filtered target compounds was compared with the corresponding reference materials. Results from GRA were imported into Origin software 2021 to generate a heat map, which was used to visualize changes in the spectrum efficiency correlation coefficient.

## 3 RESULTS AND DISCUSSION

### 3.1 Guang Chenpi could rescue spleen deficiency‐induced fatigue

**FIGURE 1:**
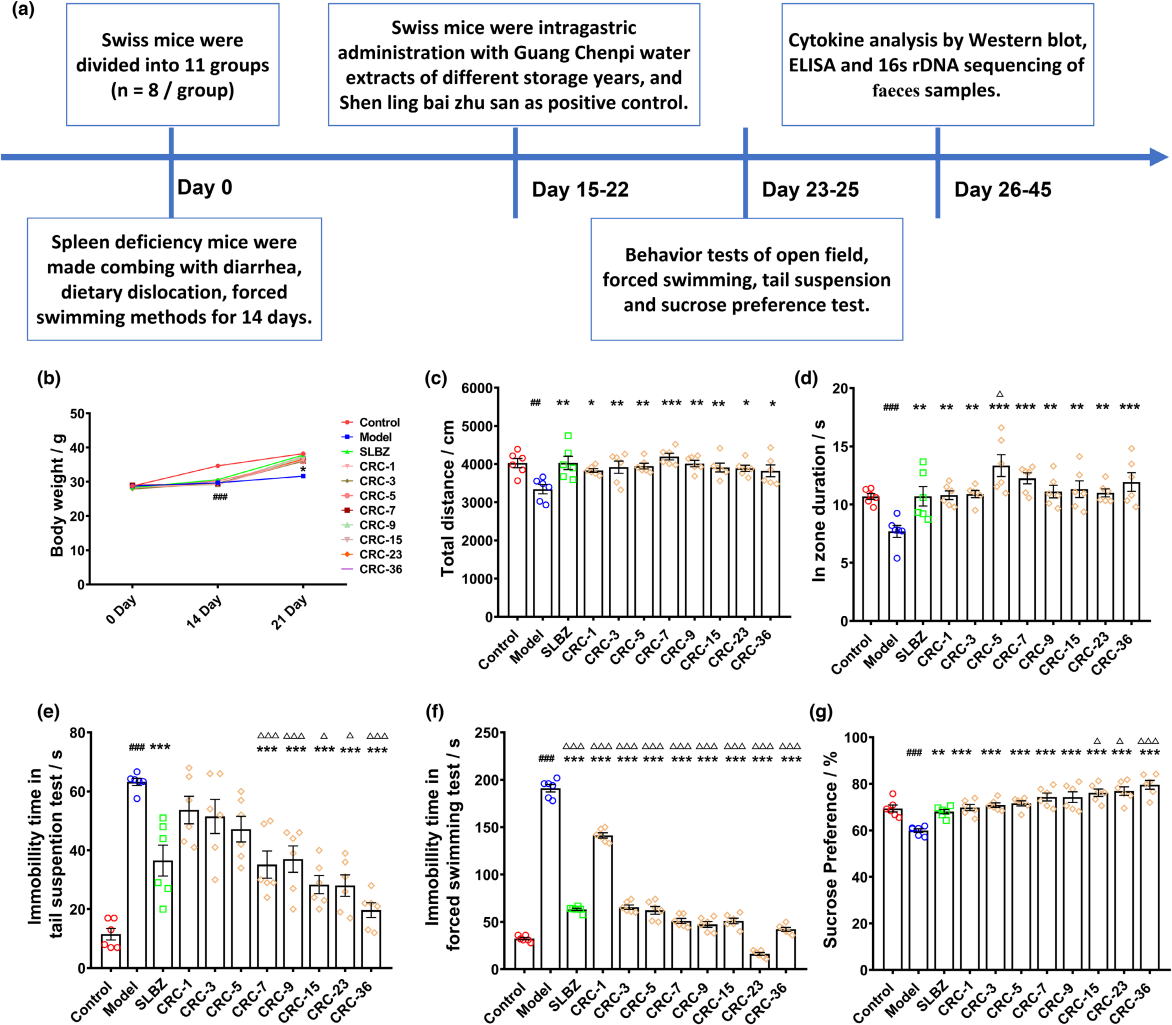
Guang Chenpi with different storage years could alleviate fatigue induced by spleen deficiency. (a) Experimental procedure. (b) Body weight loss was reversed by Guang Chenpi. (c, d) Total distance and inzone duration of each group in the open‐field test. (e) Immobility time of each group in the tail‐suspension test. (f) Immobility time in the forced swimming test. (g) Sucrose preference test for each group. (#*p* < .05, ##*p* < .01, ###*p* < .001, compared with control group; *p < .05, **p < .01, ***p < .001, compared with model group; △*p* < .05, △△*p* < .01, △△△*p* < .001, compared with CRC‐1Y group). All data are presented as mean ± SEM.

Guang Chenpi is the dry pericarp from the fruit of CRC obtained from the Guangdong province in China. “Chen Jiu Zhe Liang” means “longer storage years and better activities.” An extended storage period is a representative approach used in traditional Chinese medicine (Fu et al., 2017). To explore the scientific mechanism of this method, we first performed a pharmacological evaluation with respect to the amelioration of spleen deficiency using Guang Chenpi samples stored for different years. Spleen deficiency is associated with Qi deficiency, digestive dysfunction, and other symptoms of fatigue in Chinese medicine theory (Wang et al., 2019). To mimic these symptoms, mice were subjected to the starvation and overeating method in combination with the *i.g*. administration of aqueous senna leaf extract and to physical exhaustion by forced swimming (Zhang et al., 2020). In agreement with previous studies, mice with spleen deficiency exhibited symptoms of fatigue and digestive dysfunction, indicating the successful establishment of the model (Zhang, Yu, Zhang, et al., 2022). The complete experimental protocols are listed in Figure 1a. After the treatment on the 7th day, the model mice had loose stools, appeared tired, had unpolished fur, did not move freely (Figure S1a), and lost weight (Figure 1b) compared with control mice, suggesting the successful establishment of the mouse model of spleen deficiency. Mice with spleen deficiency traveled a shorter total distance and exhibited lesser in‐zone duration in the OFT (*p* < .01, *p* < .001), suggesting that the spontaneous movement of model mice had decreased (Figure 1c,d). In the tail‐suspension and forced swimming tests, model mice exhibited more immobility time than control mice (*p* < .01, *p* < .01), which was indicative of fatigue (Figure 1e,f). In the sucrose preference test, the preference rate of model mice was lower than that of control mice (*p* < .01) (Figure 1g). Treatment with SLBZ or CRC could rescue behavioral dysfunction in mice with spleen deficiency. The immobility time of mice in the CRC groups with long storage years (7–36 years) was shorter compared with that of mice in the CRC groups with short storage years (1–5 years) (*p* < .01). This finding indicated that Guang Chenpi extracts with different storage years could rescue spleen deficiency‐induced fatigue and that the fatigue‐ameliorating effect increased with an increase in storage years.

### 3.2 Guang Chenpi could ameliorate spleen deficiency–induced digestive dysfunction

**FIGURE 2:**
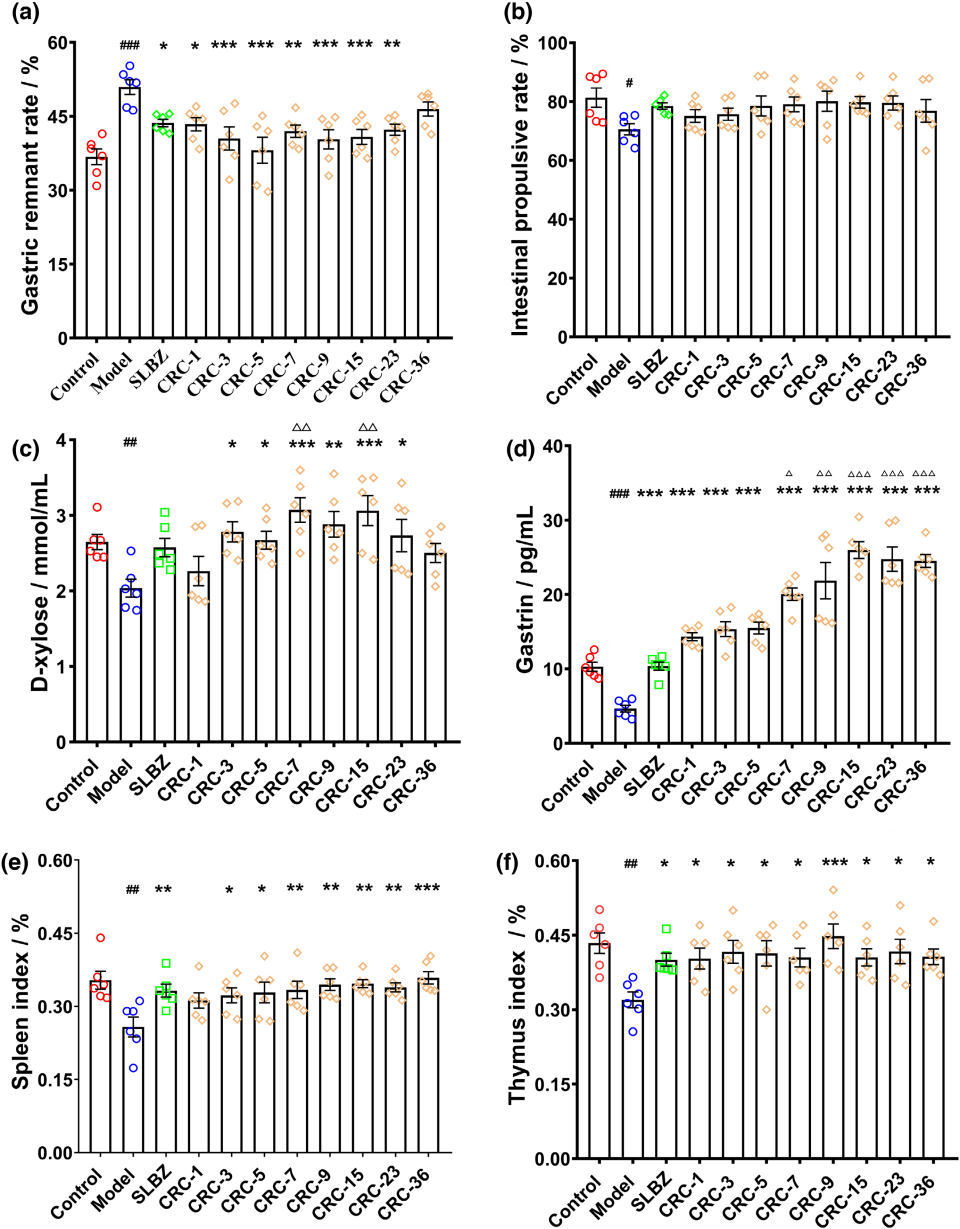
Guang Chenpi with different storage years could ameliorate gastric and immune organ dysfunction. (a, b) The effect of Guang Chenpi on gastric remnants and emptying. (c) The effect of Guang Chenpi on serum D‐xylose levels. (d) The effect of Guang Chenpi on serum gastrin levels. (e, f) Spleen and thymus indices of each group. (#*p* < .05, ##*p* < .01, ###*p* < .001, compared with control group; *p < 0.05, **p < 0.01, ***p < .001, compared with model group; △*p* < .05, △△*p* < .01, △△△*p* < .001, compared with CRC‐1Y group). All data are presented as mean ± SEM.

We further evaluated the amelioration of digestive dysfunction, gastric residual rate, and gastric emptying rate after administration of different Guang Chenpi samples. As shown in Figure 2a,b, the gastric residual rate in spleen deficiency mice was significantly higher (*p* < .01). The intestinal propulsive rate was lower than that in mice with spleen deficiency, but the difference was not significant. After the administration of Guang Chenpi extracts, intestinal propulsive rates were nearly identical to those of the control mice, indicating that Guang Chenpi could promote the digestive ability of mice with spleen deficiency. D‐xylose is a pentose that is not detected in the blood. D‐xylose levels in the blood can be used to indirectly evaluate the absorption function of intestinal mucosa after administering a certain dose of D‐xylose solution (You et al., 2020). Serum D‐xylose levels reflect the absorption ability of the small intestine. As shown in Figure 2c, D‐xylose levels in model mice were significantly lower than those in control mice (*p* < .01), suggesting absorption dysfunction in the intestine. Guang Chenpi samples with different storage years could increase D‐xylose levels, especially when samples stored for longer than 7 years were used. GAS secreted by the G cells in the gastric antrum is an important index that indicates the physiological function of the gastrointestinal tract (Niu et al., 2020). Gastrin is another important index for measuring the physiological function of the gastrointestinal tract. Guang Chenpi samples could restore lowered gastrin levels induced by spleen deficiency (Figure 2d). Guang Chenpi samples stored for more than 7 years could promote gastrin secretion. Spleen deficiency can also influence the immune system. Thus, we determined the organ indices of the spleen and thymus. As shown in Figure 2e,f, these indices were significantly decreased in model mice (*p* < .01) and rescued after the administration of Guang Chenpi samples. However, there were no significant differences in outcomes after the use of within‐year and aged samples.

The ameliorative effect increased with an increase in storage years compared with that of the within‐year sample. In addition, there were no significant differences among all indices, except D‐xylose and GAS levels, when Guang Chenpi extracts from different storage years were used. Additionally, aged Guang Chenpi samples could influence the digestive system by ameliorating gastric and intestinal function much more than samples aged for only 1–5 years.

### 3.3 Guang Chenpi with different storage years could modulate the abundance and composition of the gut microbiota

Intestinal integrity is altered by dysfunction resulting from certain diseases (Shi et al., 2020). To evaluate the intestinal permeability and ameliorative effects of Guang Chenpi in mice with spleen deficiency, levels of the linking protein occludin were determined and its distribution in the colon was monitored (Figure S1b). Occludin expression in colon tissues in the model group was significantly lower than that in the control group (*p* < .05). Occludin expression was significantly higher in the CRC‐36Y group than in the model group (*p* < .05); however, there were no significant differences in expression between the CRC‐1Y and model groups. In addition, H&E staining of colon tissues from the model group showed a thinner intestinal wall, which could be rescued by treatment with Guang Chenpi (Figure S1c). These findings showed that CRC‐1Y, CRC‐5Y, and CRC‐36Y could repair the intestinal barrier in mice with spleen deficiency. The gut microbiota plays an important role in good health, and its dysfunction has been associated with many diseases (Qian et al., 2021; Tung et al., 2018). To further explore the correlation between gut microbiota and Guang Chenpi samples (different storage years) in mice with spleen deficiency, 16S rRNA gene sequencing was used to analyze the fecal samples of mice after the oral administration of Guang Chenpi stored for 5 and 36 years. Therefore, feces samples from mice treated with CRC‐5Y and CRC‐36Y were chosen for gut microbiota analysis.

**FIGURE 3:**
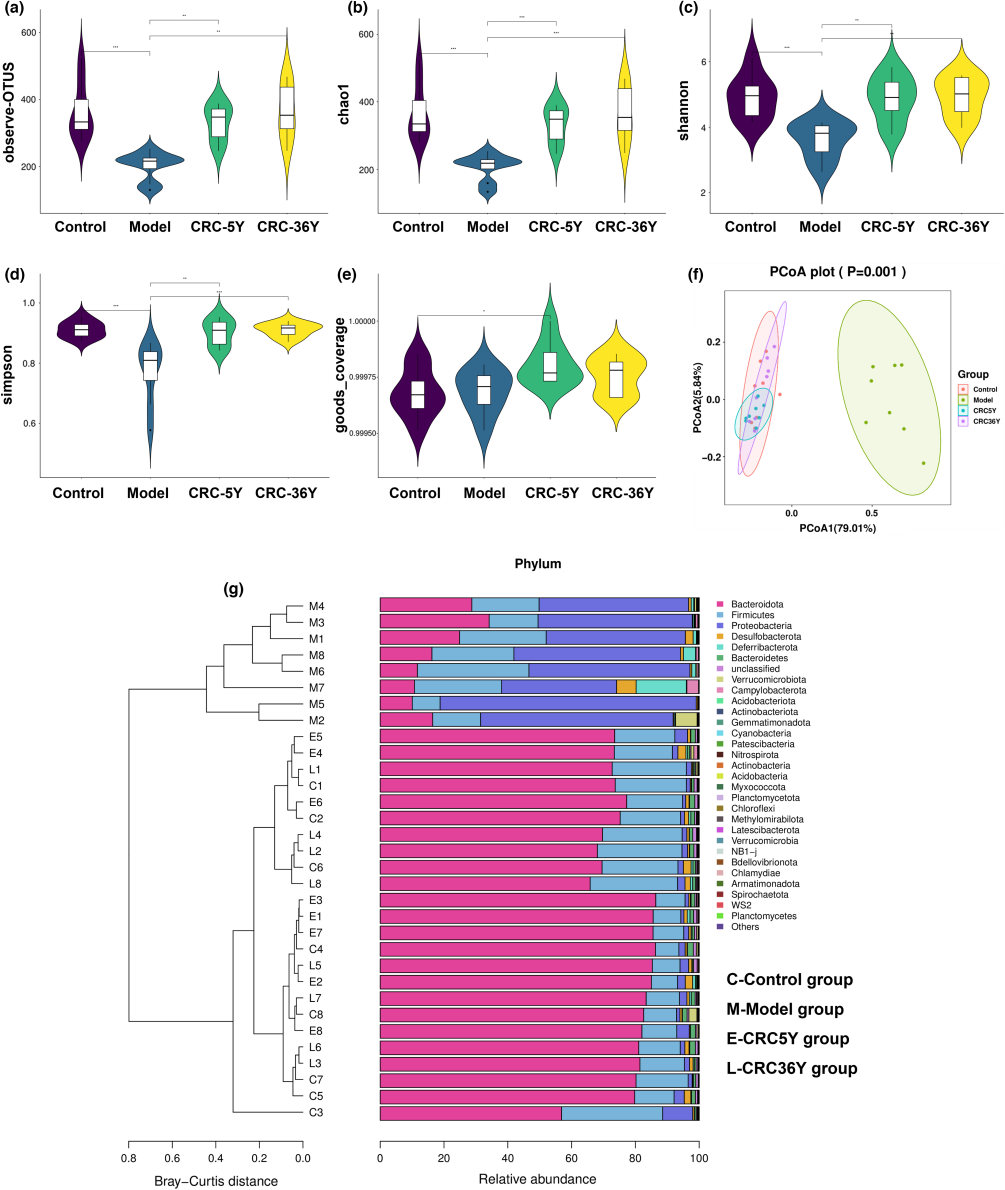
Gut microbial abundance at the phylum level. (a–e) Alpha diversity based on observing OTUs, the Chao1 index, the Shannon index, Simpson index and goods_coverage index. (f) PCoA plots were constructed using the weighted UniFrac PCoA method. (g) Composition and relationship of intestinal microbiota in the Control, Model, CRC5Y, and CRC36Y groups. (*p < .05, **p < .01, ***p < .001). All data are presented as mean ± SEM.

A key metric for analyzing microbial diversity was diversity, which was used to determine Chao1, Shannon, Simpson, and goods‐coverage diversity measures. According to this information, the model group showed a lower level of alpha diversity compared with the control and CRC groups (Figure 3a–e), suggesting that Guang Chenpi treatment might increase the abundance of gut microbes. Principal coordinate analysis (PCoA) of weighted UniFrac distance matrices was used to analyze the bacterial communities of these groups. According to PCoA's first principle, the model group is separated from the control and CRC groups. It was revealed that bacteria belong to the Bacteroidetes, Firmicutes, and Proteobacteria phyla, which were the most dominant in mice's colons (62.93%, 17.74%, and 14.73%) (Figure 3f). As shown in Figure 3g, different groups had different dominant phyla. A model group was dominated by proteobacteria, firmicutes, and bacteroidetes, whereas CRCs and controls were dominated by bacteroidetes and firmicutes. Using the unweighted pair group method with the arithmetic mean tree, almost all mice in the model CRC groups were found to be clustered together, distinctly separated from the model group, and clustered together with the control group (Figure 3g). These findings showed that similar alpha and beta diversity was observed in the intestinal microbiota in the control and CRC groups. The abundance and composition of the gut microbiota in the model group changed markedly.

**FIGURE 4:**
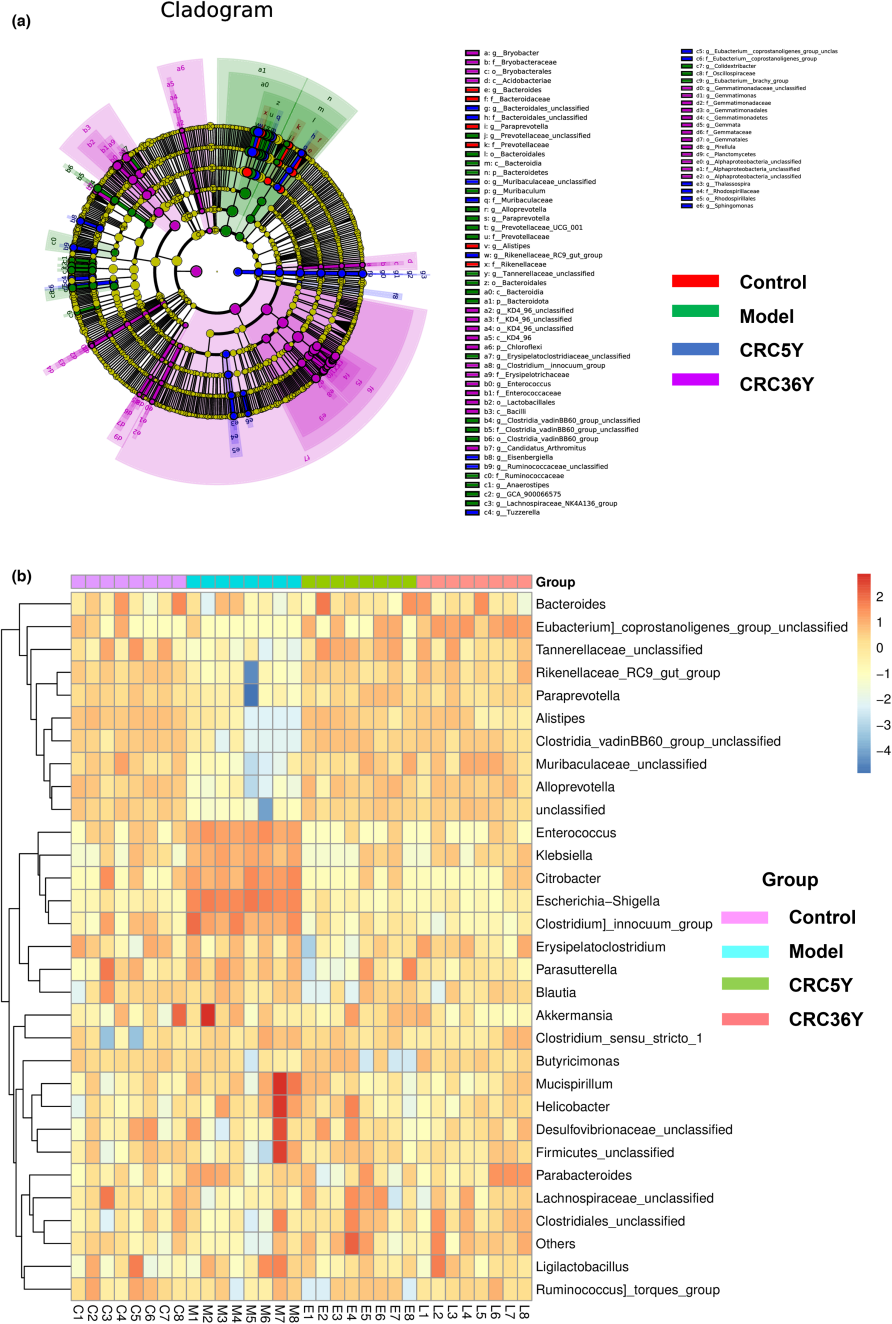
Differences in the composition of the gut microbiota among different groups. (a) Taxonomic cladogram obtained from LEfSe analysis, representing the abundance of microbiota. (b) Heatmap plot depicting the normalized abundance of each microbiota genus in the Control, Model, CRC5Y, and CRC36Y groups. (*p < .05, **p < .01, ***p < .001). All data are presented as mean ± SEM.

Furthermore, linear discriminant analysis (LDA) effect size (LEfSe) was used to determine the differential microbial composition among groups. According to a cladogram that records the hierarchical taxonomic structure of the gut microbiota, there are notable differences in the phylogenetic distributions among the phyla and genera among the groups (Figure 4a). By using a logarithmic cutoff point of 3.0, the LDA score has been calculated to indicate that the genera *Enterococcus*, *Gemmata*, *Citrobacter*, *Escherichia_Shigella*, and *Klebsiella* were significantly overrepresented in the model group. Administration of aged Guang Chenpi extracts downregulated these levels. *Bacteroides*, *Muribaculaceae_unclassified*, *Alloprevotella*, *Paraprevotella*, *Alistipes*, *Eisenbergiella*, and *Colidextribacter* were downregulated in the model group but enriched in the CRC groups. The heat map further demonstrated the differences in gut microbiota among the three groups (Figure 4b and Figure S2).

**FIGURE 5:**
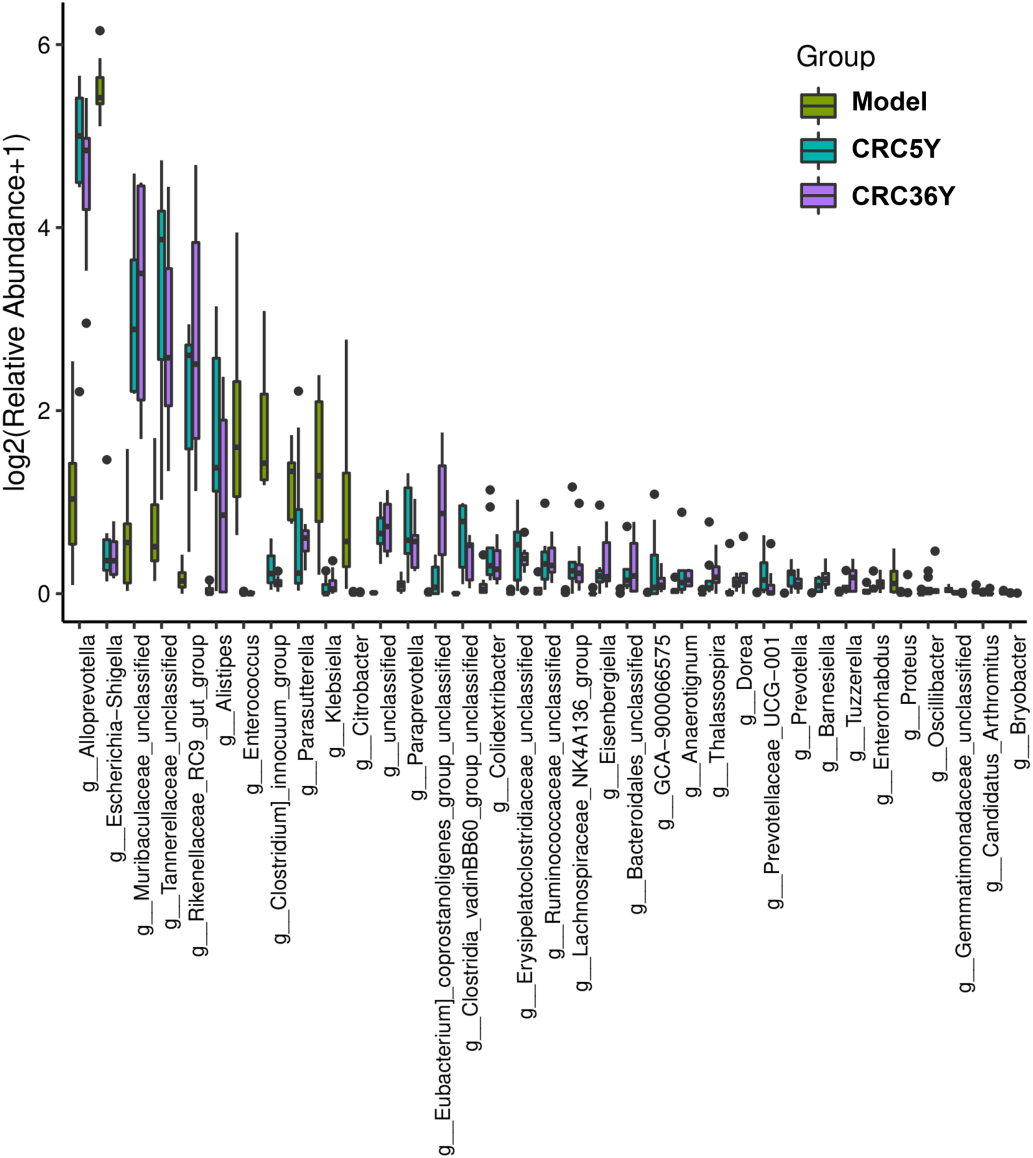
Comparison of the gut microbiota among the Model, CRC5Y, and CRC36Y groups. Differences in the relative abundance of gut microbiota at the genus level (Mann–Whitney *U* test). The box presents the 95% Cls, and the line inside denotes the median.

The relative abundance of the genera *Enterobacter*, *Enterococcus*, *Citrobacter*, and *Escherichia‐Shigella* was significantly lower in the model group compared with that in the CRC group, whereas that of *Alloprevotella* and *Tannerellaceae_unclassified* increased after CRC treatment in mice with spleen deficiency (Figure 5). Some of these results overlap with those from LEfSe, suggesting that these microbes may be the target of CRC. To further compare the microbiota abundance between the CRC‐5Y and CRC‐36Y groups, the biomarker in LEfSe showed that *Eubacterium_coprostanoligenes_group_unclassified* was significantly higher in the CRC‐36Y than in the CRC‐5Y group (Figure S2). There were significant differences in abundance between the CRC‐5Y and CRC‐36Y groups (*p* < .05). These data showed that aged Guang Chenpi extracts could restore intestinal wall integrity as well as enteric microbial richness and diversity in mice with spleen deficiency.

### 3.4 Characteristics of Guang Chenpi with different storage years determined using LC‐Q‐Exactive Orbitrap/MS

**FIGURE 6:**
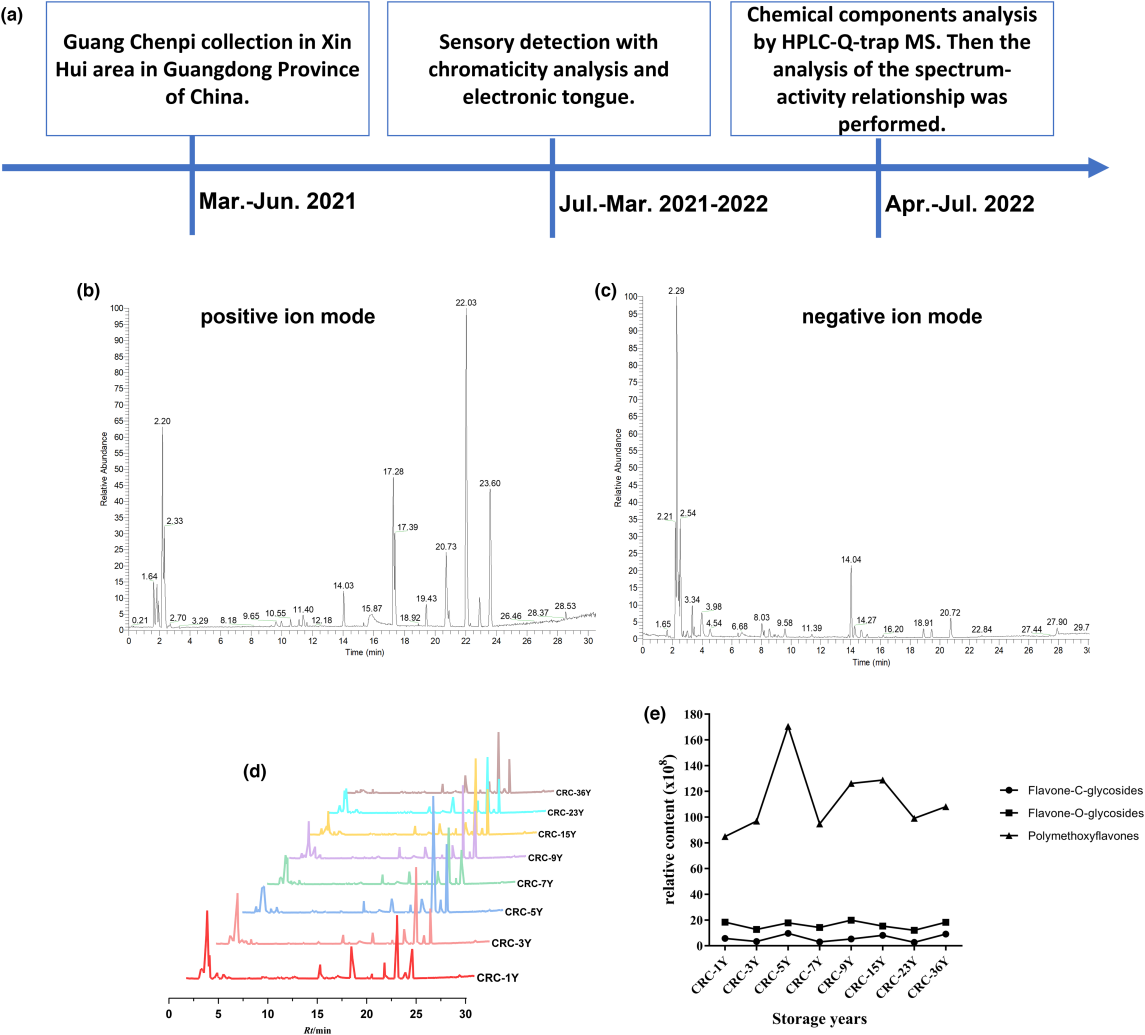
Analysis of the chemical components of Guang Chenpi water extracts using LC‐Q‐Orbitrap/MS. (a) Procedure of the spectrum‐effect analysis. (b, c) Typical total ion chromatograms of samples by LC‐QOrbitrap/MS in positive and negative ion modes. (d) The fingerprints of different samples. (e) Contents of different flavones in different samples.

To compare the chemical constituents among Guang Chenpi of different storage years and their association with bioactivities, LC‐Q‐Exactive Orbitrap/MS was used to analyze the chemical constituents of Guang Chenpi water extract. The procedure is shown in Figure 6a. The total ion current from the positive and negative ion modes reveals different chemical constituents (Figure 6b,c). The similarities and differences can also be observed in the representative chromatograms of different storage years (Figure 6d).

A total of 53 compounds were identified from Guang Chenpi samples. Quasi‐molecular ions exhibited equivalence to [M+H]^+^ and [M−H]^−^ and were unambiguously or tentatively identified through alignment with accurate molecular weights within the mass accuracy of <10 ppm. Detailed information on the chemical compounds and their distribution in Guang Chenpi samples with different storage years is shown in Table 1. Analysis of the chemical composition of Guang Chenpi revealed a change in the chemical contents with storage years. Flavonoids account for 89% of the total compounds, and 42 common peaks were identified in all samples, including alkaloids (1, 25), organic acids (21), limonin (22, 24, 39), and flavonoids (2–20, 23, 26–28, 40–53). These peak numbers were listed in Table 1. Flavanone aglycone, flavone‐c‐glycosides, flavone‐O‐glycosides, and polymethoxyflavones (PMFs) were the flavonoids in the samples. Changes in the contents of chemical constituents, including 3′,4′,5,5′,7‐pentamethoxyflavone, 3′,4′,3,5,6,7‐hexamethoxyflavone, nobiletin isomer, and orientin, were observed with an increase in storage years. Furthermore, the PMF content of samples from different storage years was compared; the levels were high during the first 5 years and decreased beyond 7 years of storage (Figure 6e). Overall, the sample stored for 5 years showed a higher flavonoid content, especially that of PMFs, compared with those stored for other time periods.

**TABLE 1:** Identification based on UPLC‐Q‐Exactive Orbitrap/MS of Citri Reticulatae Pericarpium with different storage years.

	Identification	RT	Molecular formula	[M+H]	[M−H]	Positive mode	Negative mode	1	3	5	7	9	15	23	36
1	Synephrine	1.92	C_9_H_13_NO_2_	168.102		150.0909 135.0675 119.0490 107.049 191.0545		+	+	+	+	+	+	+	+
2	Lucenin‐2	7.08	C_27_H_30_O_16_	611.1602	609.1464	341.0653 473.1063 575.1362 593.1524 539.1128	399.0722 369.0617 489.1039 519.1149 429.0819	+	+	+	+	+	+	+	+
3	Vicenin‐2	8.18	C_27_H_30_O_15_	595.165	593.1514	557.1564 457.1122 559.1420 541.1340	353.0665 383.0774 297.0769 503.1181 473.110	+	+	+	+	+	+	+	+
4	Stellarin‐2	8.54	C_28_H_32_O_16_	625.1755	623.1617	607.1609 505.1293 487.1229	383.0774 503.1195 533.1327 413.0878	+	+	+	+	+	+	+	+
5	Diosmetin‐6,8‐di‐C‐glucoside	8.93	C_28_H_32_O_16_	625.1755	623.1617	607.1624 505.1293 487.1220 439.1005	383.0771 503.1327 533.1327 413.0878	+	+	+	+	+	+	+	+
6	Isoorientin	9.72	C_21_H_20_O_11_		447.0923		357.0610 327.0505	+	+	+	−	+	+	−	+
7	Orientin	10.42	C_21_H_20_O_11_		447.0925		357.0610 327.0505	+	−	+	−	+	+	−	+
8	Rutin	11.29	C_27_H_30_O_16_		609.1458		301.0303	+	−	+	−	−	+	−	+
9	Lonicerin	11.44	C_27_H_30_O_15_		593.1502		285.0398	+	+	+	+	+	+	+	+
10	Diosmetin	12.05	C_22_H_22_O_11_		461.1085		314.0658 298.0475 371.0763 313.0721	+	+	+	−	+	+	−	+
11	Diosmetin‐6‐C‐glucoside	12.43	C_22_H_22_O_11_	463.1209	461.1091	313.0692 343.0798 367.0783 353.0986	298.0479 341.0665 371.0780 313.0719	+	+	+	+	+	+	+	+
12	PMF‐glycoside	12.95	C_28_H_32_O_16_		623.1600		315.0503 300.0267	+	+	+	+	+	+	+	+
13	Isopyrenin	13.08	C_23_H_24_O_12_		491.1181		371.0765 328.0574 360.0479 356.0533	+	+	+	+	+	+	+	+
14	Rhoifolin	13.10	C_27_H_30_O_14_	579.1687		433.1113 271.0591		+	+	+	+	+	+	+	+
15	Narirutin/naringin	13.24	C_27_H_32_O_14_	581.1845	579.1718	237.0749 153.0179	271.0612 151.0026	+	+	+	+	+	+	+	+
16	Diosmin	13.38	C_28_H_32_O_15_	609.1792	607.1676	463.1210 301.0696 286.0461	299.0554 284.0320	+	+	+	+	+	+	+	+
17	Neodiosmin	13.68	C_28_H_32_O_15_	609.1803	607.1648	301.0697 463.1232 286.0462	299.0559 284.0324	+	+	+	+	+	+	+	+
18	Hesperidin	14.04	C_28_H_34_0_15_	611.1947	609.1823	303.0854 263.0550 195.0283 177.0542 153.0178	301.0717 286.0481 242.0579	+	+	+	+	+	+	+	+
19	Homoeriodictyol	14.04	C_16_H_14_0_6_	303.0854		177.0542 153.0178		+	+	+	+	+	+	+	+
20	Neohesperidin	14.41	C_28_H_34_0_15_	611.1957	609.1823	303.0859 195.0291 177.0547 153.0183	301.0718 286.0486 242.0574	+	+	+	+	+	+	−	+
21	Ferulic acid	14.21	C_10_H_10_0_4_		191.9452		134.0359 178.0265	+	+	+	+	+	+	+	−
22	Isoobacunoic	15.12	C_26_H_32_0_8_	473.2153		161.059 495.0495		+	+	+	+	+	+	+	+
23	Poncinrin	16.38	C_28_H_34_0_14_	595.2009	593.1877	287.0910 153.0181 161.0596	285.0767 270.0533	+	+	+	+	+	+	+	+
24	Isolimonic acid	16.54	C_26_H_32_O_9_	487.1968		347.1858		+	+	+	−	+	+	+	+
25	CitrusinIII	16.99	C_36_H_53_0_9_N_7_	728.3951		474.2321 377.1815 700.3987		+	+	+	+	+	+	+	+
26	Monhydroxy‐trimethoxyflavanone	17.23	C_18_H_16_0_6_	329.1007		314.0771 299.0539 271.0593 181.0127 153.0180		+	+	+	+	+	+	+	+
27	7‐Hydroxy‐5,6,8,3′,4′‐pentamethoxy flavone	18.407	C_19_H_18_O_6_	389.1215		374.0951 359.0746 341.0633 197.0076		+	+	+	+	+	+	+	+
28	5‐hydroxy‐6,7,8,3′,4′‐pentamethoxyflavone	19.21	C_19_H_18_O_6_	389.1214		359.0744 341.0641 374.0974 197.0077		+	+	+	+	+	+	+	+
29	5,6,7,3′,4′‐pentamethoxyflavanone	19.32	C_19_H_18_O_8_	375.1425		211.0595 196.0360 150.0308		+	+	+	+	+	+	+	+
30	Hesperetin	19.37	C_16_H_14_0_6_		301.0712		286.0473 151.0023	+	+	+	+	+	+	+	+
31	2′,3′,4′,5,7‐Pentamethoxyflavone	19.42	C_20_H_20_O_7_	373.1267		343.0799 358.1039 315.0848 153.0179 357.0956 313.0683		+	+	+	+	+	+	+	+
32	7‐Hydroxy‐5,6,8,4′‐pentamethoxyflavone	19.61	C_19_H_18_O_7_	359.1109		344.0888 329.0645 301.0693		+	+	+	+	+	+	+	+
33	3′‐Hydroxy‐5,6,7,8,4′‐pentamethoxyflavone	19.88	C_20_H_20_O_8_	389.1216		359.0749 344.0512 374.0970 211.0225		+	+	+	+	+	+	+	+
34	5,7,8,3′,4′,5′‐Hexamethoxyflavone	20.17	C_21_H_22_O_8_	403.1375		373.0904 327.0846 388.1130 330.0731 358.0663		+	+	+	+	+	+	+	+
35	5,7,3′,4′‐Tetramethoxyflavone	20.22	C_19_H_18_O_6_	343.1169		327.0860 328.0923 312.0623 299.0909 283.0608		+	+	+	+	+	+	+	+
36	Monhydroxy‐pentamethoxyflavanone	20.52	C_20_H_22_O_8_	391.1379		241.0704 226.0470 211.0236 183.0288		+	+	+	+	+	+	+	+
37	Isosinensetin	20.73	C_20_H_20_O_7_	373.1267		343.0799 315.0849 153.0184 357.0954 329.0997		+	+	+	+	+	+	+	+
38	5,6,7,4′‐Pentamethoxyflavone	20.91	C_19_H_18_O_6_	343.1163		313.0698 285.0749 328.0934 299.0905 153.0180		+	+	+	+	+	+	+	+
39	Limonin	21.51	C_26_H_30_O_8_		469.1853		229.1219	+	+	+	+	+	+	+	+
40	5,7,8,3′,4′‐Pentamethoxyflavanone	21.52	C_19_H_18_O_8_	375.1427		211.0595 196.0363 168.0415		+	+	+	+	+	+	+	+
41	5,6,7,3′,4′,5′‐Hexamethoxyflavone	21.62	C_21_H_22_O_8_	403.1375		373.0908 388.1130 358.0666 330.0721		+	+	+	+	+	+	+	+
42	Nobiletin	22.04	C_21_H_22_O_8_	403.1372		373.0905 388.1154 313.0696 358.0660 330.0718		+	+	+	+	+	+	+	+
43	Nobiletin isomer	22.32	C_21_H_22_O_8_	403.1376		373.0908 388.1130 358.0666 330.0721		−	−	−	+	−	+	+	−
44	5,6,7,8,3′,4′‐Hexamethoxyflavanone	22.22	C_21_H_24_O_8_	405.1517		241.0698 226.0464 211.0233		+	+	+	+	+	+	+	+
45	5,7,8,4′‐Tetramethoxyflavone	22.30	C_20_H_20_O_7_	343.1167		313.0693 285.0746 328.0915 327.0856 299.0905 153.0177		+	+	+	+	+	+	+	+
46	3′,4′,3,5,6,7‐Hexamethoxyflavone	22.77	C_21_H_22_O_8_	403.1377		373.0908 315.0496 183.0288 330.0709 211.0237		−	+	−	−	−	−	+	+
47	3,5,6,7,8,3′,4′‐Heptemethoxyflavone	22.90	C_22_H_24_O_9_	433.1482		403.1004 418.1233 385.0896 345.0589		+	+	+	+	+	+	+	+
48	3‐Methoxynobiletin	23.17	C_22_H_24_O_9_	433.1473		403.1009 418.1229 385.0907 345.0597		+	+	+	+	+	+	−	+
49	3′,4′,5,5′,7‐Pentamethoxyflavone	23.59	C_20_H_20_O_7_	373.1274		343.0799 358.1034 328.0576 325.0686 315.0852		+	−	+	−	+	+	−	+
50	Tangeretin	23.71	C_20_H_20_O_7_	373.1272		343.0805 358.1046 297.0743 325.0698 315.0862		+	+	+	+	+	+	+	+
51	Hexamethoxyflavone	24.66	C_21_H_22_O_8_	403.1377		373.0910 330.0725 183.0290 211.0236		+	+	+	+	+	+	+	+
52	Monhydroxy‐pentamethoxyflavanone	24.76	C_20_H_20_O_8_	389.1218		374.0976 359.0746 341.0639		+	+	+	+	+	+	+	+
53	Citromitin	25.38	C_21_H_24_O_8_	405.1530		241.0702 191.0702 151.0753		+	+	+	+	+	+	+	+

The flavone and PMFs in Guang Chenpi exert several effects such as antioxidant, digestion‐promoting, cough‐ and asthma‐relieving, and anticancer (Bai et al., 2017; Benavente‐Garcia & Castillo, 2008; Keshari et al., 2016; Roohbakhsh et al., 2015). Especially, citrus PMFs can attenuate the metabolic syndrome by regulating the gut microbiome and amino acid metabolism (Zeng et al., 2020). Thirty‐one metabolites have been identified to distinguish Citri Reticulatae Chachi Pericarpium in samples from different storage years. The metabolite levels increase when stored for 3–10 years and decrease after 15–30 years of storage (Luo et al., 2019). Polymethoxyflavones tangeretin and 3,5,6,7,8,3′,4′‐heptamethoxyflavone have been reported as the most important PMFs in distinguishing various Guang Chenpi according to storage years, which contributed most to anticancer effects (Tao et al., 2022). The existing studies also focus on the differences in harvest time, such as Guang Chenpi collected in the early months containing higher concentrations of bioactive flavonoids and exhibiting potent anti‐lipase activity (Zeng et al., 2018). Nonvolatile components are considered the characteristic components for identifying Guang Chenpi harvest at different stages (Zheng et al., 2021). Targeted metabolomics technology combined with total antioxidant capacity analysis showed that flavones in CRP with different harvest times were endowed with different efficacy and usage (Liang et al., 2022). As for the anti‐spleen deficiency bioactivity identified in this research, some PMFs were also identified as the most important compounds in distinguishing storage years, especially the 5‐year ones. As a result, flavone compound content was mostly different among Guang Chenpi samples stored for various years.

### 3.5 Spectrum‐effect relationship between chemical fingerprints and amelioration of spleen deficiency

**FIGURE 7:**
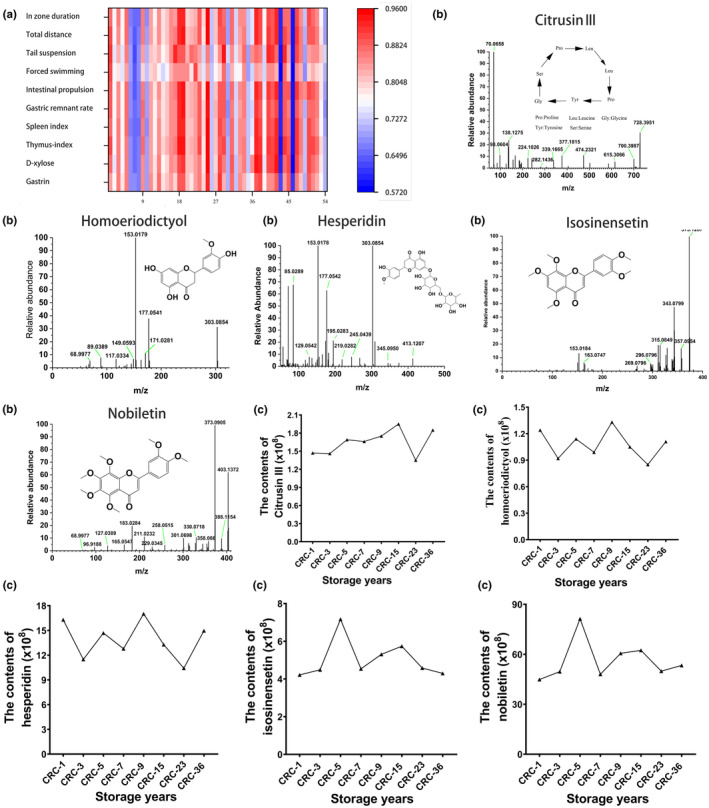
Spectrum‐effect correlation between the relative content of the common peaks and bioactivity indices using gray relational grade. (a) Heatmap of spectrum–effect correlation analysis created using gray relational grade. (b) Structures of the most correlated compounds based on gray relational grade. (c) Relative contents of the five compounds in different samples.

A total of 53 peaks and ten activity indices were used for gray relational analysis (GRA). The mean coefficient of the 29 peaks was >0.8, indicating a strong correlation, and the mean correlation coefficient of the other 13 peaks was between 0.7 and 0.8, indicating a moderate correlation (Table 2). These findings indicate that Guang Chenpi contains multiple compounds that exert synergistic effects. Peaks 18, 15, 14, 30, and 35 were the top five ranked peaks that correlated with the activity of ameliorating spleen deficiency and, hence, may be the major bioactive components of Guang Chenpi (Figure 7a). The structures of these five compounds (alkaloid, flavonoid, and PMFs) are identified in Figure 7b. There was a change in the levels of chemical contents after different storage years. As shown in Figure 7c, with an increase in storage years, peak areas of 18, 30, and 35 tended to increase, whereas peak areas of 15 and 14 did not change significantly.

**TABLE 2:** The correlation coefficient between indexes of spleen deficiency and the content of common peaks.

NO.	Identification	Correlation coefficient										Mean correlation coefficient	Ranking
		Gastrin	D‐xylose	Thymus Index	Spleen index	Gastric remnant rate	Intestinal propulsion	Forced swimming	Tail suspension	Total distance	In zone duration		
1	Synephrine	0.831	0.873	0.907	0.881	0.884	0.891	0.902	0.913	0.896	0.874	0.8852	11
2	Lucenin‐2	0.776	0.752	0.756	0.76	0.773	0.762	0.757	0.748	0.762	0.76	0.7606	41
3	Vicenin‐2	0.864	0.866	0.86	0.86	0.846	0.853	0.782	0.825	0.85	0.862	0.8468	25
4	Stellarin‐2	0.786	0.763	0.768	0.773	0.777	0.775	0.753	0.748	0.774	0.772	0.7689	39
5	Diosmetin‐6,8‐di‐C‐glucoside	0.865	0.867	0.867	0.875	0.88	0.875	0.837	0.858	0.873	0.874	0.8671	19
6	Isoorientin	0.676	0.677	0.679	0.672	0.679	0.681	0.701	0.7	0.684	0.671	0.682	49
7	Orientin	0.686	0.65	0.65	0.656	0.652	0.648	0.643	0.634	0.648	0.656	0.6523	51
8	Rutin	0.667	0.663	0.663	0.658	0.667	0.667	0.687	0.687	0.668	0.656	0.6683	50
9	Lonicerin	0.853	0.846	0.871	0.866	0.873	0.869	0.795	0.839	0.866	0.866	0.8544	21
10	Diosmetin	0.694	0.689	0.693	0.687	0.693	0.696	0.724	0.705	0.698	0.685	0.6964	48
11	Diosmetin‐6‐C‐glucoside	0.845	0.834	0.841	0.834	0.841	0.838	0.799	0.814	0.836	0.834	0.8316	30
12	PMF‐glycoside	0.78	0.779	0.775	0.77	0.776	0.779	0.812	0.802	0.779	0.768	0.782	36
13	Isopyrenin	0.876	0.859	0.85	0.853	0.84	0.848	0.776	0.818	0.846	0.854	0.842	28
14	Rhoifolin	0.784	0.778	0.797	0.789	0.783	0.785	0.733	0.778	0.787	0.79	0.7804	38
15	Narirutin/naringin	0.809	0.803	0.821	0.815	0.806	0.809	0.757	0.797	0.81	0.815	0.8042	33
16	Diosmin	0.841	0.847	0.845	0.853	0.853	0.854	0.82	0.85	0.852	0.851	0.8466	26
17	Neodiosmin	0.796	0.839	0.86	0.847	0.852	0.857	0.845	0.933	0.862	0.843	0.8534	22
18	Hesperidin	0.886	0.903	0.936	0.926	0.932	0.928	0.853	0.891	0.928	0.926	0.9109	3
19	Homoeriodictyol	0.884	0.911	0.945	0.93	0.935	0.937	0.854	0.889	0.937	0.928	0.915	2
20	Neohesperidin	0.813	0.812	0.809	0.806	0.809	0.814	0.848	0.822	0.814	0.803	0.815	32
21	Ferulic acid	0.791	0.808	0.825	0.817	0.823	0.824	0.785	0.87	0.828	0.816	0.8187	31
22	Isoobacunoic	0.835	0.841	0.838	0.833	0.843	0.84	0.902	0.858	0.841	0.827	0.8458	27
23	Poncinrin	0.866	0.861	0.899	0.888	0.891	0.892	0.805	0.839	0.894	0.887	0.8722	15
24	Isolimonic acid	0.8	0.784	0.783	0.795	0.798	0.786	0.745	0.74	0.786	0.797	0.7814	37
25	CitrusinIII	0.922	0.957	0.949	0.96	0.946	0.954	0.835	0.867	0.948	0.96	0.9298	1
26	Monhydroxy‐trimethoxyflavanone	0.725	0.716	0.716	0.722	0.717	0.718	0.717	0.709	0.717	0.722	0.7179	45
27	7‐Hydroxy‐5,6,8,3′,4′‐pentamethoxy flavone	0.813	0.801	0.808	0.812	0.821	0.808	0.773	0.782	0.81	0.813	0.8041	34
28	5‐Hydroxy‐6,7,8,3′,4′‐pentamethoxyflavone	0.909	0.901	0.89	0.901	0.888	0.894	0.8	0.845	0.89	0.903	0.8821	13
29	5,6,7,3′,4′‐Pentamethoxyflavanone	0.829	0.868	0.901	0.877	0.883	0.888	0.876	0.947	0.892	0.871	0.8832	12
30	Hesperetin	0.711	0.703	0.71	0.697	0.687	0.7	0.708	0.729	0.7	0.697	0.7042	47
31	2′,3′,4′,5,7‐Pentamethoxy‐flavone	0.87	0.888	0.88	0.879	0.869	0.881	0.821	0.844	0.88	0.877	0.8689	18
32	7‐Hydroxy‐5,6,8,4′‐pentamethoxy‐flavone	0.719	0.715	0.731	0.73	0.729	0.728	0.76	0.744	0.73	0.73	0.7316	43
33	3′‐Hydroxy‐5,6,7,8,4′‐pentamethoxyflavone	0.852	0.836	0.853	0.849	0.839	0.845	0.771	0.819	0.842	0.851	0.8357	29
34	5,7,8,3′,4′,5′‐Hexamethoxyflavone	0.827	0.86	0.862	0.854	0.862	0.863	0.848	0.876	0.866	0.848	0.8566	20
35	5,7,3′,4′‐Tetramethoxyflavone	0.745	0.721	0.717	0.724	0.715	0.717	0.697	0.689	0.717	0.723	0.7165	46
36	Monhydroxy‐pentamethoxy‐flavanone	0.767	0.847	0.759	0.745	0.735	0.749	0.728	0.852	0.749	0.745	0.7676	40
37	Isosinensetin	0.892	0.944	0.931	0.929	0.909	0.93	0.834	0.88	0.927	0.929	0.9105	4
38	5,6,7,4′‐Pentamethoxyflavone	0.905	0.926	0.911	0.905	0.887	0.907	0.825	0.865	0.904	0.905	0.894	8
39	Limonin	0.771	0.753	0.755	0.759	0.763	0.752	0.728	0.733	0.755	0.757	0.7526	42
40	5,7,8,3′,4′‐Pentamethoxyflavanone	0.847	0.897	0.919	0.908	0.905	0.917	0.85	0.923	0.919	0.901	0.8986	6
41	5,6,7,3′,4′,5′‐Hexamethoxyflavone	0.851	0.9	0.896	0.894	0.881	0.892	0.847	0.919	0.892	0.889	0.8861	11
42	Nobiletin	0.895	0.936	0.932	0.926	0.907	0.928	0.83	0.871	0.926	0.926	0.9077	5
43	Nobiletin isomer	0.633	0.604	0.597	0.599	0.599	0.598	0.574	0.594	0.601	0.601	0.6	52
44	5,6,7,8,3′,4′‐Hexamethoxyflavanone	0.833	0.875	0.91	0.883	0.89	0.894	0.873	0.931	0.899	0.878	0.8866	9
45	5,7,8,4′‐Tetramethoxyflavone	0.885	0.9	0.892	0.881	0.865	0.883	0.805	0.84	0.881	0.881	0.8713	17
46	3′,4′,3,5,6,7‐Hexamethoxyflavone	0.613	0.579	0.596	0.596	0.597	0.593	0.573	0.593	0.593	0.595	0.5928	53
47	3,5,6,7,8,3′,4′‐Heptemethoxyflavone	0.857	0.907	0.914	0.912	0.893	0.911	0.856	0.896	0.911	0.906	0.8963	7
48	3‐Methoxynobiletin	0.811	0.848	0.859	0.859	0.846	0.859	0.83	0.862	0.861	0.853	0.8488	23
49	3′,4′,5,5′,7‐Pentamethoxyflavone	0.712	0.717	0.721	0.72	0.711	0.852	0.678	0.689	0.711	0.722	0.709	44
50	Tangeretin	0.891	0.898	0.904	0.896	0.879	0.898	0.804	0.842	0.894	0.896	0.8802	14
51	Hexamethoxyflavone	0.851	0.882	0.898	0.882	0.873	0.886	0.827	0.855	0.886	0.878	0.8718	16
52	Monhydroxy‐pentamethoxyflavanone	0.824	0.847	0.87	0.848	0.857	0.861	0.802	0.852	0.864	0.847	0.8472	24
53	Citromitin	0.777	0.785	0.794	0.777	0.767	0.779	0.779	0.816	0.781	0.773	0.7828	35

Spectrum‐effect analysis revealed that besides the uncommon peaks, the common peaks from different samples, especially homoeriodictyol, hesperidin, isosinensetin, and nobiletin, correlated well with spleen deficiency amelioration. Although the levels of these compounds were mostly unchanged, they correlated most with activity differences associated with storage years. Previous studies have shown that hesperidin and nobiletin can ameliorate brain, liver, and heart dysfunction.

Although interesting results were obtained after multivariate statistical analysis in this study, some problems remain to be resolved. From the aspect of identification of chemical compounds, the high‐throughput method should be used to determine differences in chemical constituents induced by storage years. Our future study will involve qualitative and quantitative analyses of the primary compounds. Moreover, the specific mechanisms of action of new compounds will be elucidated, which will provide more clarity on the mechanism of action of “Chen Jiu Zhe Liang” in Guang Chenpi.

## 4 CONCLUSIONS

In this study, LC‐Q‐Exactive Orbitrap/MS profiles and in vivo anti‐spleen deficiency effects were combined to determine the spectrum‐effect relationship of Guang Chenpi with different storage years. Aged Guang Chenpi showed better effects in ameliorating spleen deficiency by regulating gut microbiota than those of within‐year samples. GRA revealed that flavonoids such as citrusin III, homoeriodictyol, hesperidin, isosinensetin, and nobiletin were the main active constituents associated with alleviating spleen deficiency. The exact pharmacological mechanism of the main compounds will be studied subsequently to explain the theory of “Chen Jiu Zhe Liang.”

## AUTHOR CONTRIBUTIONS

**Xiaoming Sun:** Conceptualization (lead); data curation (lead); methodology (lead); validation (lead); writing – original draft (lead). **Haidan Deng:** Data curation (equal); investigation (equal); methodology (equal); validation (equal). **Baojun Shan:** Data curation (equal); investigation (equal); methodology (equal); validation (equal). **Yunqi Shan:** Methodology (supporting); project administration (supporting); writing – review and editing (supporting). **Jiaying Huang:** Methodology (supporting). **Xinshu Feng:** Methodology (supporting). **Xiaomin Tang:** Methodology (supporting); project administration (supporting). **Yuewei Ge:** Investigation (supporting); methodology (supporting); project administration (supporting); software (supporting). **Peiran Liao:** Investigation (supporting); validation (supporting). **Quan Yang:** Conceptualization (lead); funding acquisition (lead); investigation (lead); methodology (lead); supervision (lead); validation (lead); writing – review and editing (lead).

## FUNDING INFORMATION

This work is supported by the Key Project at the Central Government level: The ability establishment of sustainable use for valuable Chinese medicine resources (2060302) and University Characteristic Innovation Project of Guangdong Province (2019KTSCX072).

## CONFLICT OF INTEREST STATEMENT

All authors agree to submit this article and declare no conflict of interest.

## Supporting information


Figure S1

Figure S2

Appendix S1

## Data Availability

The data that support the findings of this study are available on request from the corresponding author.
