# Supplementary figures and images for: Flavonoids contribute most to discriminating aged Guang Chenpi (Citrus reticulata ‘Chachi’) by spectrum‐effect relationship analysis between LC‐Q‐Orbitrap/MS fingerprint and ameliorating spleen deficiency activity
Source: Food Sci Nutr. 2023 Sep 21;11(11):7039–60. doi: 10.1002/fsn3.3629 (PMC10630847; doi:10.1002/fsn3.3629)

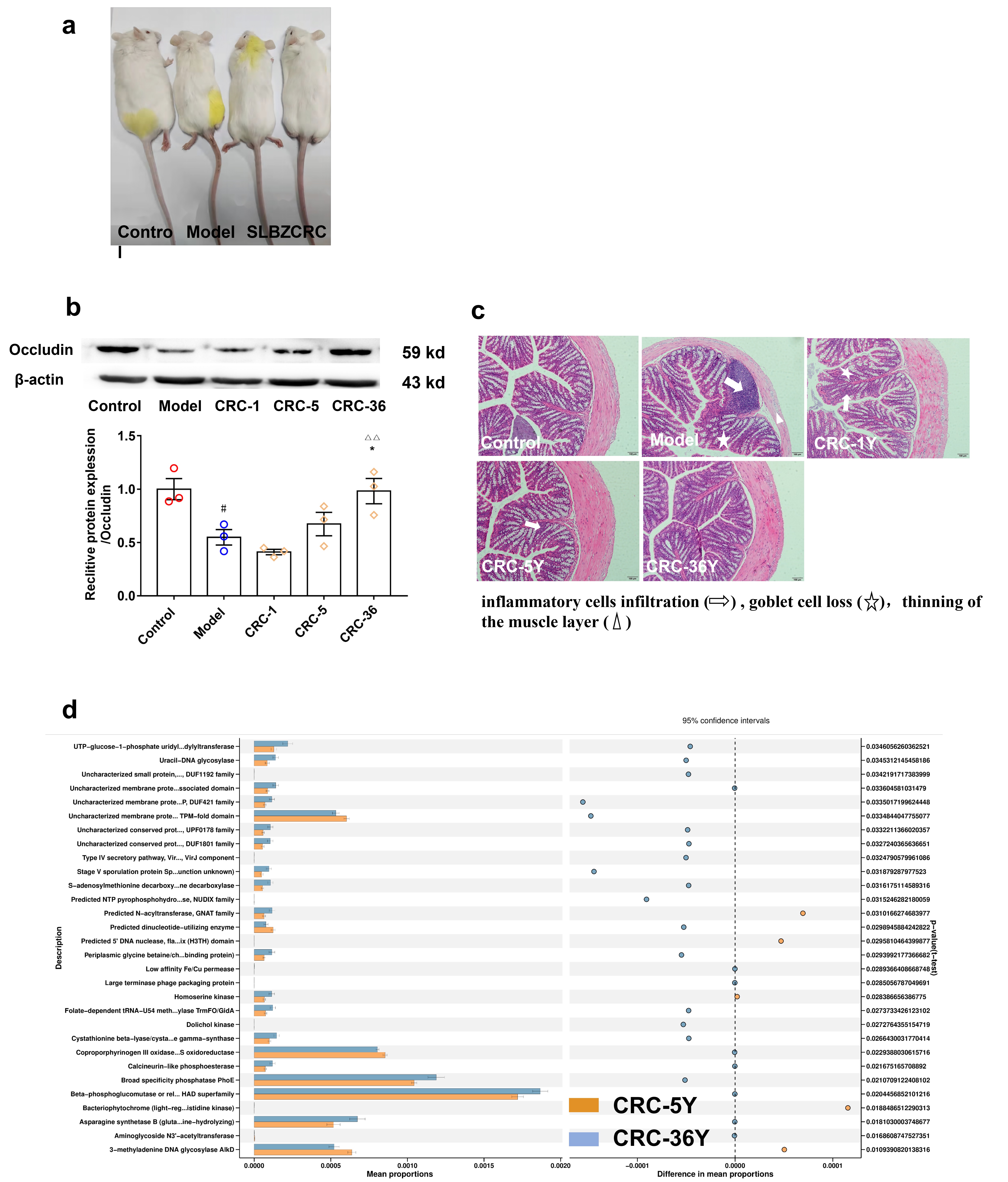

Supplement: Supplementary file 1 — Figure S1 [file FSN3-11-7039-s001.jpg]

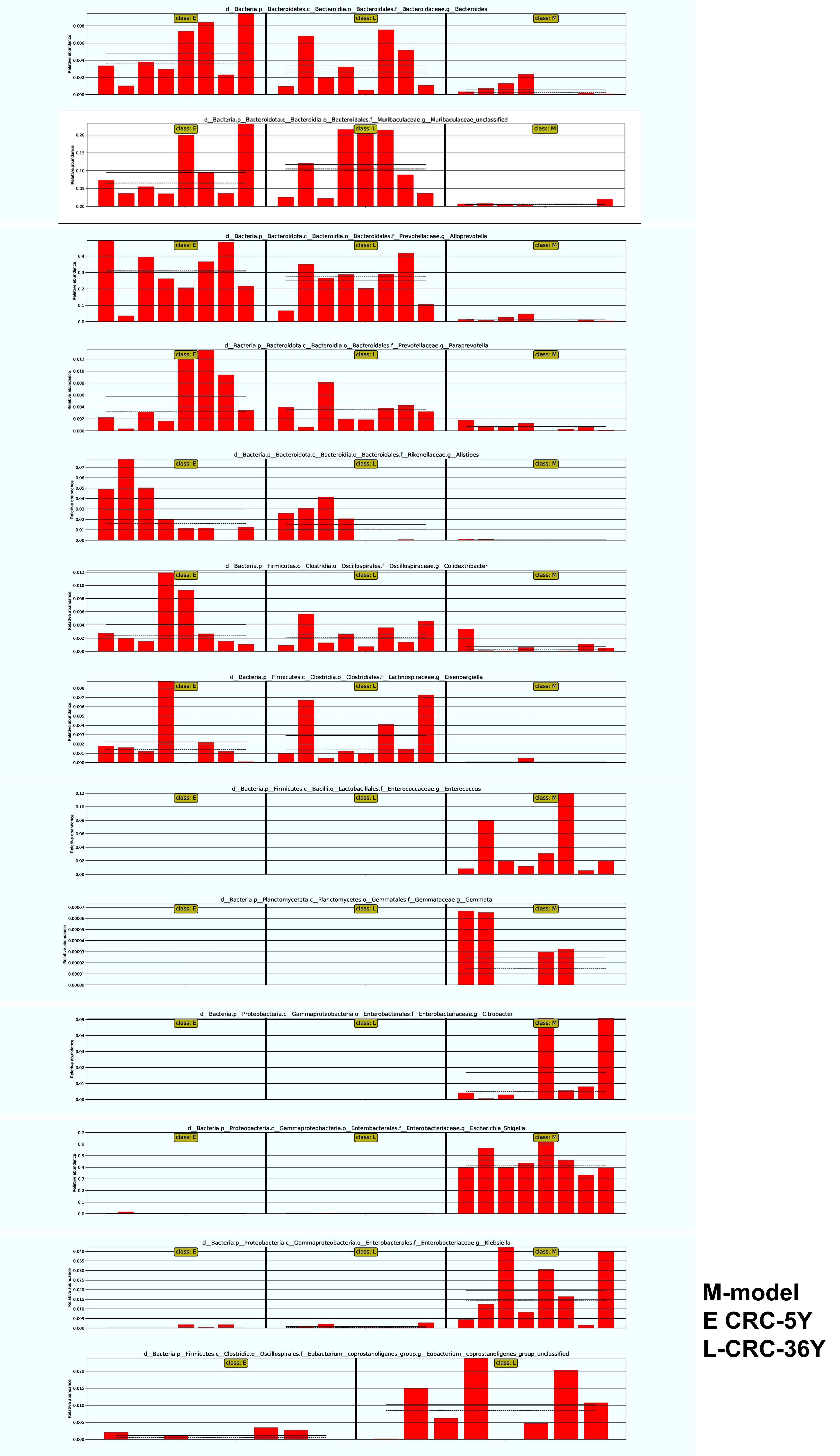

Supplement: Supplementary file 2 — Figure S2 [file FSN3-11-7039-s003.tif]
