# Supplementary material for: Flavonoids contribute most to discriminating aged Guang Chenpi (Citrus reticulata ‘Chachi’) by spectrum‐effect relationship analysis between LC‐Q‐Orbitrap/MS fingerprint and ameliorating spleen deficiency activity
Source: Food Sci Nutr. 2023 Sep 21;11(11):7039–60. doi: 10.1002/fsn3.3629 (PMC10630847; doi:10.1002/fsn3.3629)
Supplement: Supplementary file 3 — Appendix S1 [file FSN3-11-7039-s002.docx]

1. Instruments

DFY-400C rocking high-speed grinder (Wenling Linda Machinery Co., Ltd.), AX224ZH analytical balance (Ohaus Instruments (Changzhou) Co., Ltd. ), L550 low-speed centrifuge (Hunan Xiangyi Laboratory Instrument Development Co., Ltd.), MulitskanMK3 microplate reader (Thermo Fisher Scientific and Technological Co., Ltd.), RM2016 pathological slicer (Shanghai Leica Instrument Co., Ltd.) and KD-P tissue slicer (Jinhua Kedi Instrument Co., Ltd., Zhejiang Province). JJ-12J dehydrator and JB-P5 embedding machine (Wuhan Junjie Electronics Co., Ltd.), OLYMPUS / BX fluorescence microscope (Guangzhou Zhongchuang Biotechnology Co., Ltd.), Tanon-5200 CE chemical luminescence gel imaging system (Shanghai Tianneng Technology Co., Ltd.), gel electrophoresis tank, protein electrophoresis wet rotation system (Bio-Rad Co., Ltd.), TS-1 rocker (Qilin Medical Instrument Factory, Haimen City), DHFSTPRP-CL24 frozen tissue grinder (Ningbo Luoshang Intelligent Technology Co., Ltd.). MINIP-2500 Microporous Centrifuge (Hangzhou Miou Instrument Co., Ltd.), KW-OPF Open-field Activity Box (Guangzhou Bite Biotechnology Co., Ltd.), U3000 Ultra High Performance Liquid Chromatography, Q Exactive Focus Mass Spectrometer (Thermo Scientific Co., USA)

2. Materials

GAS ELISA kit (Wuhan Aidi Biotechnology Co., Ltd., 202104) ; D-xylose kit (Nanjing Jiancheng Bioengineering Institute, 20210823) ; D-xylose and 4 % paraformaldehyde (both purchased from Guangzhou Binxin Biotechnology Co., Ltd., batch number : C12285705, 70100900) ; HE staining solution (servicebio, batch number : G1005). RIPA lysis buffer (Beijing Reagan Biotechnology Co., Ltd., batch number : PS0013), SDS-PAGE gel preparation kit, BCA protein concentration determination kit (Biotime biotechnology graduate student, batch number : P0012A, P0012S), rabbit anti-mouse Occludin primary antibody (Abcam Company, USA, batch number : gr3383643-13), chemiluminescence substrate, secondary antibody goat anti-rabbit IgG, rabbit anti-mouse β-actin primary antibody (Cell Signaling Technology company, USA, BL520B, 7074S, 7074). Chromatographic grade acetonitrile, methanol (Thermo Scientific Company, USA, 212213,207899), senna leaf (Foshan Zhongtian Chinese Herbal Pieces Co., Ltd.), Shenling Baizhu Powder Granules (Shanxi Huakang Pharmaceutical Co., Ltd., Z14020399).
